# A Rare Mullerian Duct Anomaly Not Included in the Classification System by the American Society for Reproductive Medicine

**DOI:** 10.1155/2013/569480

**Published:** 2013-03-21

**Authors:** Shereene J. Brown, Shawky Z. A. Badawy

**Affiliations:** Department of Obstetrics and Gynecology, State University of New York Upstate Medical University, 736 Irving Avenue, Syracuse, NY 13210, USA

## Abstract

This is a case report of a 37-year-old female with a uterine septum (two cavities), a normal single fundal contour, two cervices, and a longitudinal vaginal septum. This is a rare finding that is not described in the current classification system by the American Society for Reproductive Medicine.

## 1. Introduction

Mullerian duct anomalies have become more easily diagnosed due to better imaging modalities over the last few decades. The incidence has been quoted as low as 0.001% and as high as 10% [1]. It is theorized that the mullerian ducts fuse around the eleventh through the thirteenth weeks in utero and that fusion and absorption are unidirectional from caudal to cephalad [1–3]. Our current classification system for uterine anomalies is based on this unidirectional theory [4]. The majority of uterine anomalies can be explained by this theory; however, there are a number of cases that do not fit this paradigm [4]. The case described here is a mullerian duct anomaly with a longitudinal vaginal septum, two cervices, a uterine septum, and a single normal fundus. This represents failure of absorption of the septum that separates the mullerian ducts after fusion.

## 2. A Case Report

37-year-old G1P0101 was referred to the Reproductive Endocrinology Division because of chronic dyspareunia and secondary infertility. She had a prior Caesarean delivery where it was discovered that she had an unspecified mullerian duct anomaly. She now had a new partner and had been trying to get pregnant for three years. She had regular cycles with normal flow. On physical examination, a vaginal septum and two cervices were observed.

**Figure 1:**
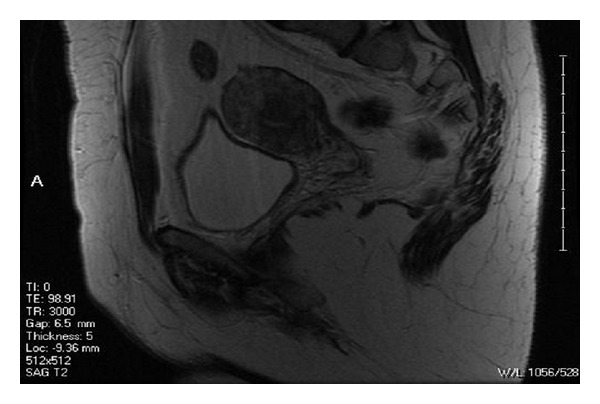
Sagittal view; note the smooth contour of the uterus.

**Figure 2:**
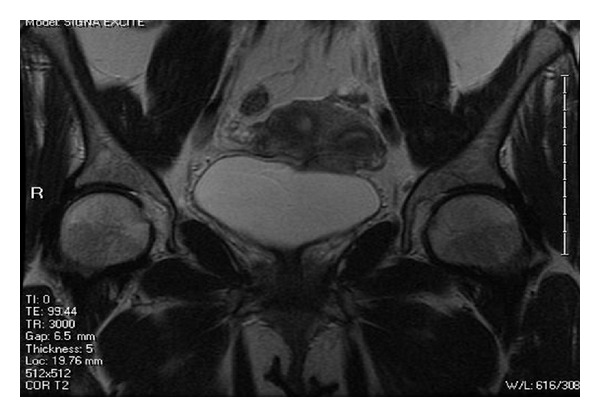
Coronal view; note the two uterine cavities, myometrium, and thick septum.

**Figure 3:**
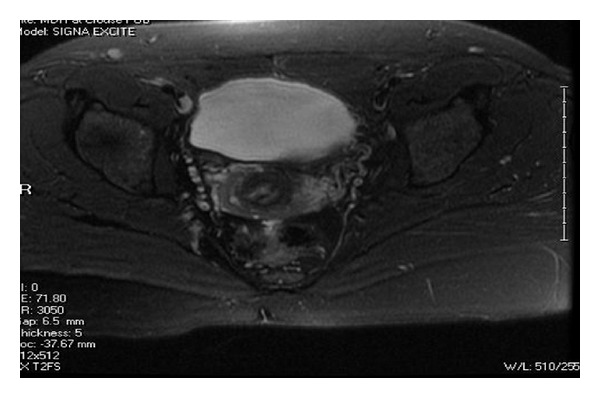
Axial view; note the cervices and two canals.

She had an MRI that showed a smooth single external uterine fundal contour without a cleft (Figure 1). There were two separate endometrial channels (Figure 2) and two upper cervical canals (Figure 3). This was thought to be consistent with a septate uterus, possible bicornuate bicollis, but not indicative of didelphys uterus. The lack of a notch between the two uterine canals suggested uterine pseudodidelphys. Another possibility was a complete septate uterus with double cervices and double vaginas. Of note, there were no renal anomalies on ultrasound.

The patient underwent examination under anesthesia, excision of the vaginal septum, and hysterosalpingogram (HSG). The uterine cavities were sounded. The left uterine cavity sounded to 3.5 inches, and the right uterine cavity sounded to 2 inches. Both uterine cavities were cannulated at the same time, and there was a septum. During the HSG, the left cervix and uterus spilled Omnipaque dye through the left fallopian tube, and, likewise, the right cervix and uterus spilled dye through the right fallopian tube. A laparoscopy was also performed demonstrating one uterine fundus, two fallopian tubes, and two ovaries. The decision was made not to remove the uterine septum because both uterine cavities were functional and she could achieve pregnancy in either cavity. Her dyspareunia improved following the removal of the vaginal septum.

On semen analysis, her husband was found to have azoospermia secondary to chemotherapy and radiation therapy. As a result of this revelation, the patient decided to proceed with artificial insemination with donor sperm.

## 3. Discussion

The etiology of mullerian duct anomalies is not well understood [4, 5]. Most defects are polygenic and multifactorial [5]. The classification system for uterine anomalies by the American Society for Reproductive Medicine (ASRM) is based on six groups [4, 6]. Greater than 90% of mullerian duct anomalies can be grouped in the ASRM classification system [5]. Of the mullerian duct anomalies, the most common is the septate uterus [5]. This case represents a uterine anomaly that is not part of the ASRM classification system.

Similar to the aforementioned case, there are cases in the literature that describe normal uteri with double cervices. Pavone et al. described a case with a septate uterus, two cervices, and a smooth fundus [1]. Shirota et al. described a case with a normal uterus, a double cervix that communicated at the internal os, and a vagina with a longitudinal septum [2]. Dunn and Hantes described a case where hysteroscopy revealed one normal cervix and the other cervix ended in a blind pouch [4]. Duffy et al. described a case with a longitudinal vaginal septum with two cervices and a normal fundus with two uterine cavities [7]. Chang et al. described five cases with uterine septum, double cervices, and vaginal septum [8]. Ribeiro et al. reported that, since 1994, approximately 40 cases of this particular anomaly have been reported [9].

The above cases are very similar in that they represent mullerian duct anomalies that do not fit into the ASRM classification system. We would like to propose the inclusion of this rare anomaly in an amended classification system by the American Society for Reproductive Medicine.
